# Linc00426 accelerates lung adenocarcinoma progression by regulating miR-455-5p as a molecular sponge

**DOI:** 10.1038/s41419-020-03259-2

**Published:** 2020-12-11

**Authors:** Hongli Li, Qingjie Mu, Guoxin Zhang, Zhixin Shen, Yuanyuan Zhang, Jun Bai, Liping Zhang, Dandan Zhou, Quan Zheng, Lihong Shi, Wenxia Su, Chonggao Yin, Baogang Zhang

**Affiliations:** 1 Experimental Center for Medicine Research, Weifang Medical University, 261053 Weifang, China; 2 Department of Pathology, School of Clinical Medicine, Weifang Medical University, 261053 Weifang, China; 3 School of Clinical Medicine, Weifang Medical University, 261053 Weifang, China; 4 College of Biological Science and Technology, Weifang Medical University, 261053 Weifang, China; 5 Department of Clinical Surgery, Affiliated Hospital of Weifang Medical University, 261053 Weifang, China; 6 College of Nursing, Weifang Medical University, 261053 Weifang, China

**Keywords:** Prognostic markers, Tumour biomarkers

## Abstract

Increasing lines of evidence indicate the role of long non-coding RNAs (LncRNAs) in gene regulation and tumor development. Hence, it is important to elucidate the mechanisms of LncRNAs underlying the proliferation, metastasis, and invasion of lung adenocarcinoma (LUAD). We employed microarrays to screen LncRNAs in LUAD tissues with and without lymph node metastasis and revealed their effects on LUAD. Among them, Linc00426 was selected for further exploration in its expression, the biological significance, and the underlying molecular mechanisms. Linc00426 exhibits ectopic expression in LUAD tissues and cells. The ectopic expression has been clinically linked to tumor size, lymphatic metastasis, and tumor differentiation of patients with LUAD. The deregulation of Linc00426 contributes to a notable impairment in proliferation, invasion, metastasis, and epithelial–mesenchymal transition (EMT) in vitro and in vivo. Mechanistically, the deregulation of Linc00426 could reduce cytoskeleton rearrangement and matrix metalloproteinase expression. Meanwhile, decreasing the level of Linc00426 or increasing miR-455-5p could down-regulate the level of UBE2V1. Thus, Linc00426 may act as a competing endogenous RNA (ceRNA) to abate miR-455-5p-dependent UBE2V1 reduction. We conclude that Linc00426 accelerates LUAD progression by acting as a molecular sponge to regulate miR-455-5p, and may be a potential novel tumor marker for LUAD.

## Introduction

Lung cancer, with a 5-year overall survival rate of 15%, is a predominant cause of cancer-related deaths among males^1^. Approximately 80% of lung cancers are non-small-cell lung cancers (NSCLCs)^2^. The two main types of NSCLCs are lung adenocarcinoma (LUAD) and lung squamous cell carcinoma^3^. Patients with LUAD often die of relapse and metastasis due to the potential of progressing to hematogenous metastasis^4^. Despite the significant progress in the treatment of LUAD in recent years, its prognosis remains poor, particularly for cases with lymph node metastasis or distant tumor metastasis. Thus, elucidating the potential mechanisms underlying the LUAD progression to identify novel targets for effective intervention is urgently needed.

Long non-coding RNAs (LncRNAs) are a class of non-protein-coding RNAs with over 200 nucleotides in length and can modulate gene expression at transcriptional or post-transcriptional level^5–7^. LncRNAs function as oncogenes or tumor suppressors by activating or silencing the expression of protein-coding genes and participating in cell proliferation, development, invasion, migration, and apoptosis^8,9^. Their expression is frequently dysregulated in multiple types of tumor such as lung cancer^10^. For example, a novel LncRNA of LUADT1 promotes LUAD proliferation via epigenetic deletion of p27^11^. The enhanced expression of LncRNA of ZXF1 facilitates the invasion and metastasis of LUAD^12^. Moreover, another lowly expressed LncRNA of RPLP0P2 is associated with poor prognosis and can abolish the cell abilities of proliferation and adhesion in LUAD^13^.

Recently, a type of LncRNAs has been classified as ceRNAs which are involved in the crosstalk among LncRNAs, mRNAs, and their shared microRNAs (miRNAs). This revealed a new regulatory mechanism by which LncRNAs and mRNAs communicate with each other by competing for the common miRNAs response element. Here, we investigated the role of the Linc00426-miR-455-5p-UBE2V1 axis in regulating the process of LUAD progression and the underlying mechanisms. Given its significance in the process of tumor progression, Linc00426 may act as a promising predictive biomarker for LUAD, and proper regulation of the Linc00426-miR-455-5p-UBE2V1 axis may be a novel synergistic strategy for inhibiting the process of tumor progression.

## Materials and methods

### LncRNAs microarray and data analyses

For LncRNAs microarray analyses, six LUAD tissue samples (three LUAD tissues with lymph node metastasis and three without) were obtained from the Affiliate Hospital of Weifang Medical University. The levels of LncRNAs were profiled using Human LncRNA Array V3.0 (Arraystar, Rockville, Maryland, USA), and analyzed by KangChen Bio-tech Inc. (Shanghai, China). The differentially expressed genes were employed for cluster analysis of samples and heat map generation.

### Human tissue specimens

This study was approved by the Ethics Committee of Weifang Medical University and written informed consents were obtained from all subjects included in the research. The tissues were obtained from the Affiliate Hospital of Weifang Medical University. Based on the literature review and our preliminary experiments, a significant level of α = 0.05, power of test = 0.9, and 144 specimens of clinical tissues (72 tissues with lymph node metastasis and 72 without) were selected. Frozen tumor samples were used for total RNA extraction using the TRIzol method. All tumor tissues were confirmed by two experienced pathologists. Clinicopathological characteristics of patients with LUAD were shown in Table 1.

**Table 1:** Correlations between Linc00426 expression and clinicopathological characteristics in 144 patients with LUAD.

Characteristics	LINC00426 expression		*p* Value
	High	Low	
**Age (years)**			
<50	38	41	0.738
≥51	34	31	
**Gender**			
Male	39	35	0.617
Female	33	37	
**Tumor size (cm)**			
≤5 cm	29	44	0.019
>5 cm	43	28	
**Tumor differentiation**			
I/II	25	41	0.012
III/IV	47	31	
**Lymphatic metastasis**			
Yes	49	23	0.000
No	23	49	
**Distant metastasis**			
Yes	47	27	0.001
No	25	45	

### Cell culture and reagents

Cells (A549, H1975, H1299, BEAS-2B, and HEK-293T) were purchased from the American Type Culture Collection. BEAS-2B and HEK293T cells were cultured in Dulbecco’s modified Eagle’s medium (DMEM), with H1299 and H1975 in RPMI-1640 medium and A549 in F12-K at 37 °C with 5% CO_2_. All cell media were supplemented with 10% fetal bovine serum (FBS).

### Western blot and immunofluorescence

Total proteins were extracted from LUAD cell lines as previously stated^14^. The following antibodies were used in this study: Vimentin (1:500), N-cadherin (1:500), E-cadherin (1:500), and UBE2V1 (1:500). The β-actin antibody was used as a control for whole-cell lysates. Antibodies were purchased from Abcam (ab92547, ab18203, ab194982, ab151725, and ab181602). A549 cells were plated on glass slides (1 × 10^4^ cells/well) and incubated for approximately 24 h at 37 °C. The cells were then washed with PBS, and harvested, followed by being subjected to 4% paraformaldehyde for fixation, 0.1% Triton X-100 for permeabilization, and serum working fluid for blocking nonspecific binding, successively. The cells were then incubated with fluorescence secondary antibodies and counterstained with 4′,6-diamidino-2-phenylindole (DAPI) the next day. Confocal laser scanning microscope (SP8, Leica, Germany) was used to obtain the fluorescence images.

### Cell proliferation

After being digested with trypsinase, cells were suspended in complete medium. Three wells for each group were seeded at 5 × 10^4^ cells/well and the cells were incubated at 37 °C with 5% CO_2_. From the second day after plating, the cell counts were detected by Celigo Image Cytometer (Celigo, Nexcelom, USA) once a day for 5 days. At each time point, cell counts of the three wells in each group were presented as the mean value. The cell growth curves of each group were plotted for five consecutive days.

### Wound-healing assays

For wound-healing assays, all cells were cultured in medium with low concentration serum (supplemented with 0.5% FBS). A scratch tool was used to generate scratches gently in the lower central part of the 96-well plates. Serum-free culture medium was used to rinse the cells gently, and culture medium with low concentration serum was added and the microscopic observation was performed at 0 h. Then, the Celigo Image Cytometer was used to analyze the images after cell migration for 24 and 32 h in the same field and photographed, respectively. Celigo Image Cytometer was used to analyze the scanning images and obtain the cell migration area.

### Cell invasion

The invasion of A549 cells was examined based on the number of cells through Matrigel-coated transwell inserts. In brief, 2 × 10^5^ cells were seeded into 24-well plate-sized inserts with Matrigel (BD Biosciences, USA). The cells were plated in serum-free medium, with the bottom chamber containing that with 10% FBS. After being incubated for both 24 and 32 h, cells without penetrating the small holes were gently removed by cotton swabs. The cells were then fixed and stained with Giemsa. Cells from five random fields of view were counted under an optical microscope (Olympus, Tokyo, Japan).

### RNA extraction and real-time quantitative PCR (RT-qPCR)

RNA level was measured by RT-qPCR. Total RNA was extracted by TRIzol reagent according to the manufacturer’s manual. M-MLV reverse transcriptase (Invitrogen, Carlsbad, CA, USA) was used for LncRNA reverse transcription. cDNA was synthesized by a MMLV first-strand cDNA synthesis kit (Promega, WI, USA). SYBR Green quantitative PCR kit (Bio-Rad, Hercules, CA, USA) was used to detect Linc00426 levels in tissues and cells. U6 and GAPDH were used as internal references for normalizing the target genes.

### Luciferase reporter assay

Linc00426 and UBE2V1 mRNA with mutant (Mut) or wild type (Wt) within miR-455-5p binding sites were cloned into upstream of the luciferase reporter. The pGL-3 Basic Luciferase Vector (Promega, USA) lacking the Linc00426 or UBE2V1 transcript inserts served as the control. The renilla reporter pRL (Promega, WI, USA) plasmid was used to normalize the transfection efficiency. Data were expressed as relative light units for luciferase normalized to renilla luciferase activity. Three independent experiments were performed.

### Vector construction and lentivirus infection

Linc00426 lentivirus was constructed by GeneChem Company (Shanghai, China). Sequences are as following: Sh-NC: TTCTCCGAACGTGTCACGT, sh-Linc00426-1#: AAGGATGGAAATACAGAACAA, sh-Linc00426-2#: TAGGTCATAATTGCTTAACTA, and sh-Linc00426-3#: CTTGTCCAATTTGTAGGGAAA. Lentivirus infection was performed according to the manufacturer’s protocol. Cells were observed under fluorescence microscope at 72 h after infection. Cells stably expressing Linc00426 shRNA were established by lentiviral transduction and puromycin selection. Stable cells were cultured for further tests. Transfection was performed with Lipofectamine 2000 (Invitrogen) according to the manufacturer’s protocol. Anti-miR-455-5p and anti-NC (7 μg) were dissolved in 2 mL MEM.

### RNA immunoprecipitation (RIP) assay

An EZMagna RIP kit (Millipore, Billerica, MA, USA) was used following the manufacturer’s protocol. A549 cells were lysed in complete RIP lysis buffer, and the cell extract was incubated with magnetic beads conjugated with specific antibodies or control IgG (Millipore) for 6 h at 4 °C. The beads were washed and then incubated with Proteinase K to detach proteins. The purified RNA was used for RT-qPCR analysis.

### Bioinformatics analysis

Target genes for miR-455-5p were identified by starbase. PPI network was built on STRING (https://string-db.org/), and cytoscape was used for seeking target genes (namely the hub gene) for miR-455-5p. The prognostic values of candidate genes were analyzed using the Kaplan–Meier Plotter (http://kmplot.com/analysis/), an online database that could assess the effects of 1,715 samples derived from ten independent datasets^15^. The correlation between mRNA expression of candidate genes and overall survival (OS) in patients with lung cancer was assessed.

### Tumor xenografts

BALB/c nude mice (4–5 weeks, *n* = 40; Charles River) were carried out with the approval of the Ethics Committee (Committee on the Use of Live Animals in Teaching and Research) of Weifang Medical University. For tumor xenograft assay, A549 cells were stably transfected with sh-NC and sh-Linc00426. Subsequently, mice were randomly grouped (n = 10) and the cells were xenografted (1 × 10^7^/100 μL) into mice after being harvested. The weight of mice and tumor volume were measured every 3 days. Tumor volume was calculated according to the formula (*x*^2^ × *y*)/2 where *x* < *y* (*x* = width; *y* = length). Thirty-five days later, the mice were killed and the tumors were stripped for further analyses.

For tumor metastasis assay, mice were randomly grouped (*n* = 10), and 1 × 10^6^ A549 cells transfected with sh-NC and sh-Linc00426 were intravenously injected into BALB/c nude mice which were sacrificed after 1 month. Lungs were removed for examination. HE staining was used to observe metastatic lesions.

### Hematoxylin-eosin (HE) staining

The lungs were collected, fixed in 4% paraformaldehyde for 24 h, dehydrated by alcohol, cleared by xylene, and imbedded in paraffin, successively. 4 mm sections were prepared and stained with HE. After being dewaxed with xylene, the sections were subjected to absolute ethanol, 95% ethanol, 80% ethanol, 75% ethanol, and distilled water, successively. The sections were then stained with hematoxylin for 1 min, washed under running water, washed by distilled water, embedded in 95% ethanol, and stained with eosin for 30 s, successively. 95% ethanol and absolute ethanol were used for dehydration, with xylene for clearing and neutral balsam for mounting. The histological changes were analyzed under an optical microscope (Olympus, Tokyo, Japan).

### Statistical analyses

All experiments were performed at least three times independently. Data were presented as the mean ± standard deviation (SD). Shapiro-Wilk test was used to analyze the normality test. All data were found in a normal distribution, and variance was similar among the groups statistically compared. All statistical analyses were carried out using SPSS18.0 software (IBM, Chicago, IL, USA). Statistical evaluation was performed using Student’s *t* test (two-tailed) between two groups. One-way analysis of variance (ANOVA) was employed to compare the difference among groups. The relationship between Linc00426 and miR-455-5p was also evaluated by Spearman correlation analysis. Statistics with *P* < 0.05 were considered as statistically different.

## Results

### Differential expression patterns of LncRNAs in LUAD tissues with and without lymph node metastasis

**Fig. 1:**
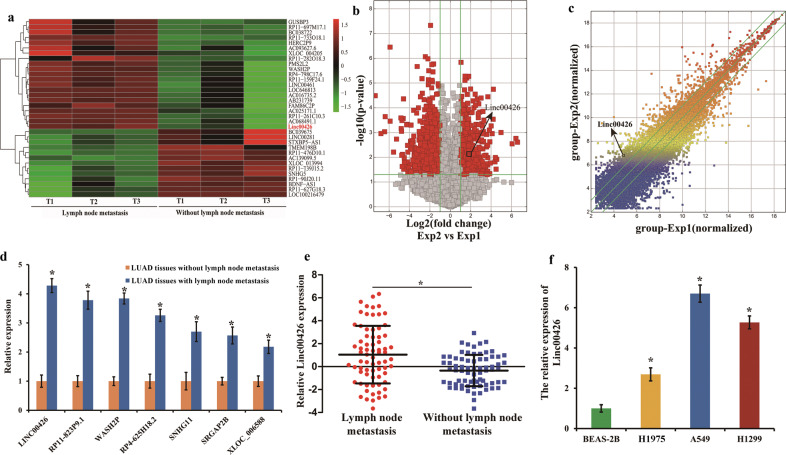
Differential expression profiles of LncRNAs in LUAD tissues with and without lymph node metastasis. **a** Distinguishable LncRNA profiling in LUAD tissues analyzed by hierarchical clustering (fold change ≥ 2; *P* < 0.05). Relative expression levels from high to low were indicated by red and green, respectively. **b** Volcano plots of LncRNAs. The red plots were differentially expressed LncRNAs with statistically significant difference (fold change ≥ 2, *P* < 0.05). **c** Variations in LncRNAs among LUAD tissues presented by scatter plots. **d** Relative expression of seven LncRNAs in LUAD tissues determined by RT-qPCR. Student’s *t* test. **e** Relative expression of Linc00426 in 72 LUAD tissues with lymph node metastasis and 72 LUAD tissues without lymph node metastasis determined by RT-qPCR. GAPDH serves as a reference gene for Linc00426. Results were presented as log2^fold^ lymph node metastasis tissues relative to without lymph node metastasis. Student’s *t* test. **f** Expression of Linc00426 in different LUAD cells and BEAS-2B cells examined by RT-qPCR. One-way ANOVA. Experiments were performed three times and data were presented as mean ± SD. ^*^P < 0.05.

Previous microarray results showed that some LncRNAs had a higher expression in LUAD tissues with lymph node metastasis. The array data have been published^16^ and the gene expression omnibus accession number is GSE115734. The microarray results showed higher expression of Linc00426 in LUAD tissues with lymphatic metastasis (Fig. 1a). The overview of scatter and volcano plots displayed similar results (Fig. 1b and c). The classification could provide some clues for exploring the role of LncRNAs. To validate the microarray results, we selected seven up-regulated LncRNAs with RNA lengths ranging from 1000 to 1100 nucleotides (Supplementary Table 1) for subsequent analyses. These LncRNAs belong to intergenic differential ones, and their expression was analyzed using RT-qPCR. The results showed that the Linc00426, derived from the seven LncRNAs, presented the most significant different expression (Fig. 1d). The data were consistent with the microarray results. We detected the expression of Linc00426 in 144 specimens of LUAD tissues by RT-qPCR, with 72 with lymph node metastasis and 72 without. The results showed that the expression of Linc00426 was more prominent in LUAD tissues with lymph node metastasis compared with those without (Fig. 1e). Based on the LncRNA microarray data of LUAD and bioinformatics algorithm, we focused on the biological function of Linc00426 and constructed a LncRNA–miRNA–mRNA network for the first time.

### Upregulation of Linc00426 in LUAD cell lines and tissues is associated with the clinicopathological features of patients with LUAD

To investigate the role of Linc00426 in LUAD, we examined its expression in normal pulmonary bronchial epithelial cells BEAS-2B and LUAD cells (H1975, A549, and H1299) by RT-qPCR. It was significantly up-regulated in all LUAD cells compared with that in normal pulmonary bronchial epithelial cells BEAS-2B (Fig. 1f). Correlation of clinicopathological features and Linc00426 expression level was investigated among LUAD tissues. No significant correlation was found between Linc00426 expression and age, gender of patients with LUAD. However, its expression was correlated with tumor size, tumor differentiation, lymph node metastasis, and distant metastasis (Table 1). Hence, Linc00426 expression might be correlated with LUAD progression and play an important role in LUAD. These data suggest that Linc00426 could be a valuable tumor marker for LUAD.

### Downregulation of Linc00426 leads to significantly inhibit proliferation, metastasis, and invasion of LUAD in vitro

**Fig. 2:**
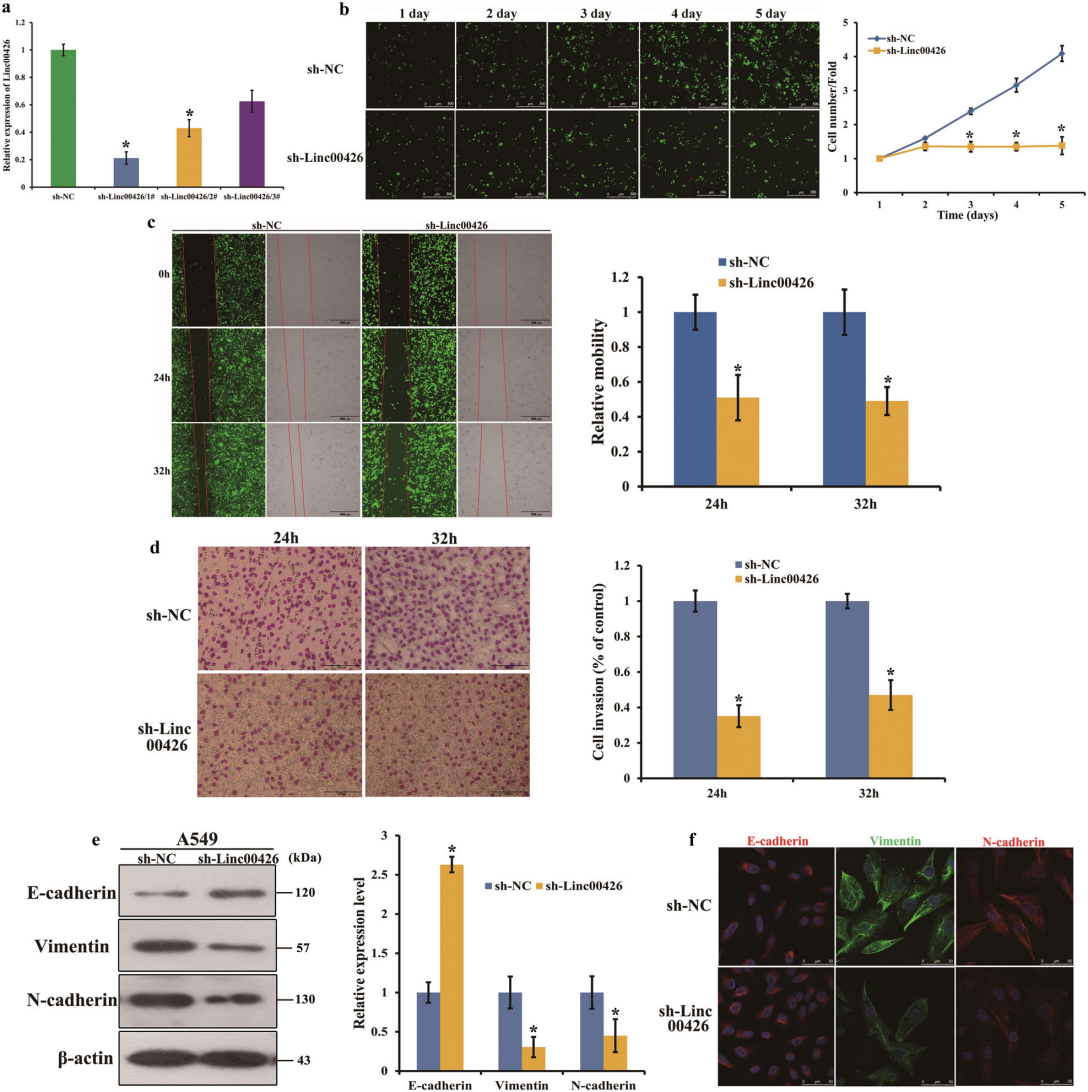
Downregulation of Linc00426 leads to significantly inhibit proliferation, metastasis, and invasion of LUAD in vitro. **a** Quantification of Linc00426 in A549 cells after infection with sh-NC and three different shRNA (sh-Linc00426-1#, sh-Linc00426-2#, and sh-Linc00426-3#). RT-qPCR was employed to examine the infection efficiency. **b** Cell proliferation was detected by Celigo Image Cytometer on the reading board once a day for 5 days. Scale bar = 500 μm. **c** Effects of Linc00426 knockdown on cellular mobility detected by wound-healing assay. Photos were taken by Celigo Image Cytometer at 0, 24, and 32 h, respectively. Scale bar = 500 μm. **d** Effects of Linc00426 knockdown on invasion ability determined using Transwell assay. Photos were taken by optical microscope at 24 and 32 h, respectively. Scale bar = 100 μm. **e** Expression levels of EMT-related markers were detected by Western blot in A549 cells infected with sh-NC and sh-Linc00426. **f** Expression of EMT-related markers in A549 cells with sh-NC and sh-Linc00426 by immunofluorescence analysis. The nuclei were counterstained using DAPI. Scale bar = 50 μm. Experiments were performed three times and data were presented as mean ± SD. ^*^P < 0.05. Student’s *t* test.

The upregulation of Linc00426 in LUAD cell lines and tissues implies that it may play a role of oncogenes in LUAD. We then examined its functional effects on LUAD cells. In this study, A549 cells were selected based on the higher expression of Linc00426 than that in BEAS-2B. We constructed stable cell lines by using a lentivirus vector to intercede Linc00426 expression in A549 cells. All siRNAs significantly decreased the expression of Linc00426. Knockdown efficiency was identified by RT-qPCR. The sh-Linc00426-1# displayed the highest efficiency, thus A549/sh-NC and A549/sh-Linc00426-1# (sh-Linc00426) were used for further studies (Fig. 2a). To confirm the role of Linc00426 in the proliferation of A549, we performed the analysis on cell proliferation. Knockdown of Linc00426 suppressed the proliferation of A549 cells compared with sh-NC (Fig. 2b). The wound-healing assay showed that Linc00426 knockdown suppressed the metastasis capacity of A549 cells compared with sh-NC (Fig. 2c). The transwell invasion assays revealed that Linc00426 knockdown inhibited the invasion of A549 cells (Fig. 2d). EMT is considered as an important mechanism involved in invasion and metastasis. In this regard, we detected whether Linc00426 could mediate EMT in LUAD cells. Immunofluorescence and Western blot analyses revealed that Linc00426 knockdown suppressed the expression of Vimentin and N-cadherin but elevated that of E-cadherin (Fig. 2e, f). These findings indicated that the downregulation of Linc00426 suppressed the proliferation, metastasis, and invasion of LUAD cells in vitro.

### Linc00426 is required for cytoskeleton rearrangement and matrix metalloproteinase expression, and downregulation of Linc00426 represses tumor growth and metastasis of LUAD in vivo

**Fig. 3:**
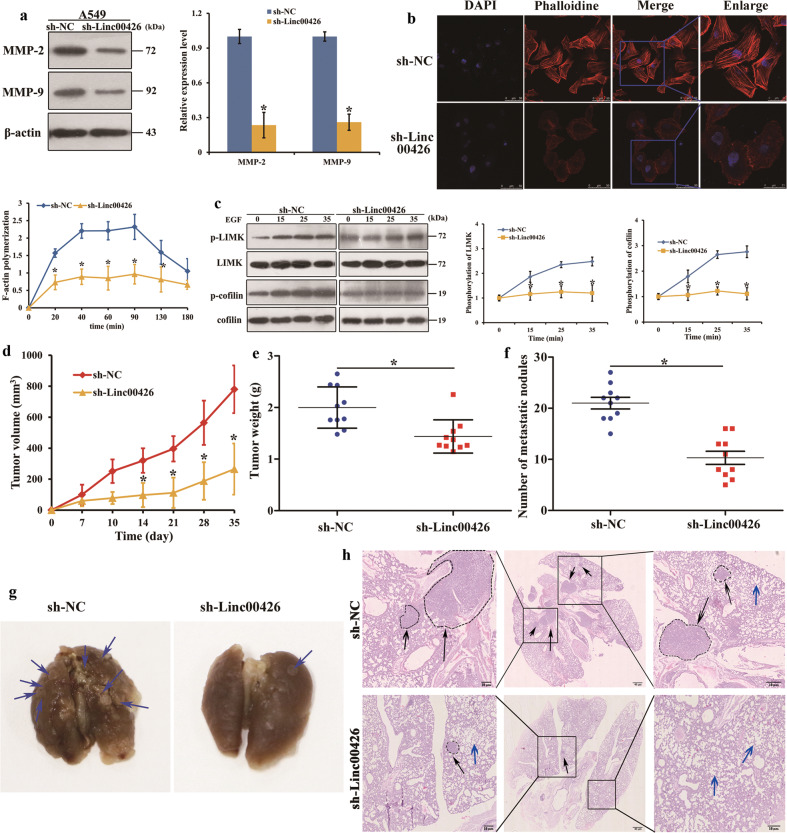
Linc00426 is required for cytoskeleton rearrangement and matrix metalloproteinase expression and downregulation of Linc00426 represses tumor growth and metastasis of LUAD in vivo. **a** The expression of MMP-2 and MMP-9 in A549 cells with sh-NC and sh-Linc00426. **b** Images of F-actin (red) and nucleus (blue) staining in A549 cells with sh-NC and sh-Linc00426. The chart shows F-actin polymerization in A549 cells with sh-NC and sh-Linc00426. Scale bar = 50 μm. **c** The expression of p-LIMK and p-cofilin in A549 cells with sh-NC and sh-Linc00426. **d** Growth curves of A549 cells with sh-NC and sh-Linc00426 derive from subcutaneous tumor xenografts. Tumor volume is measured with time duration. **e** Tumor weight is measured after surgical dissection. **f** Number of metastatic nodules in different tissue slices. **g** Bright-field imaging (arrows indicate metastases) and counting of metastatic nodules. **h** H&E staining and number of metastatic foci in the lungs injected with sh-NC and sh-Linc00426 cells. The metastatic focus is indicated with dotted line and black arrows; The alveolar is indicated with blue arrows. Scale bar = 40 μm and 10 μm. ^*^P < 0.05. Student’s *t* test.

The deregulation of matrix proteases, including matrix metalloproteinases (MMPs), is a common means for cancer to remodel the ECM, particularly in the initial phases of tumor cell dissemination and cancer development progression^17^. Cytoskeleton rearrangement can promote the migration and invasion of non-small cell lung cancer cells^18^. Thus, we detected the effects of Linc00426 on MMPs expression and cytoskeleton rearrangement in LUAD cells. MMP-2 and MMP-9 play an important role in tumor development progression by promoting migration and invasion. The Western blot data revealed that the downregulation of Linc00426 reduced the levels of MMP-2 and MMP-9 in A549 cells (Fig. 3a). F-actin polymerization is essential for the invasion of malignant tumor. We employed the analysis of F-actin polymerization to determine the effects of Linc00426 deletion on the invasion capacity of LUAD cells. Data demonstrated polymerization and notable reduction for F-actin in A549/sh-NC and A549/sh-Linc00426 cells, respectively (Fig. 3b). Hence, Linc00426 played a vital part in cytoskeleton rearrangement. LIMK and cofilin are two essential proteins involved in the regulation of F-actin polymerization^19^. Then we tested the phosphorylation level changes of LIMK and cofilin. Data showed that the deletion of Linc00426 expression restrained the phosphorylation levels of LIMK and cofilin, and the cytoskeleton was eliminated (Fig. 3c). Overall, Linc00426 is required for cytoskeleton rearrangement and MMPs expression. Based on the data from in vitro experiments, the effects of Linc00426 on LUAD tumorigenesis in vivo were investigated. A549/sh-Linc00426 or A549/sh-NC was injected subcutaneously into BALB/c nude mice. The results showed that less A549 cells with Linc00426 knockdown developed into tumors than control vector cells did. Meanwhile, the volume and weight of tumors derived from A549/sh-Linc00426 cells (*n* = 10) were notably decreased compared with those from A549/sh-NC ones (*n* = 10) (Fig. 3d). The abrogation of Linc00426 was sufficient to impede orthotopic tumorigenesis of A549 cells in vivo (Fig. 3e). Then, we examined the metastasis behavior changes in primary tumor models after subcutaneous injection of infective A549 cells. The number of metastatic nodules in the lung of nude mice with stable Linc00426 knockdown was substantially reduced compared with that in the control vector group (Fig. 3f, g). Histopathological analysis by HE staining confirmed lung metastatic foci (Fig. 3h). The results suggest that the deregulation of Linc00426 represses the tumorigenesis and metastasis of LUAD cells in vivo.

### Linc00426 acts as a ceRNA by competitive binding to miR-455-5p

**Fig. 4:**
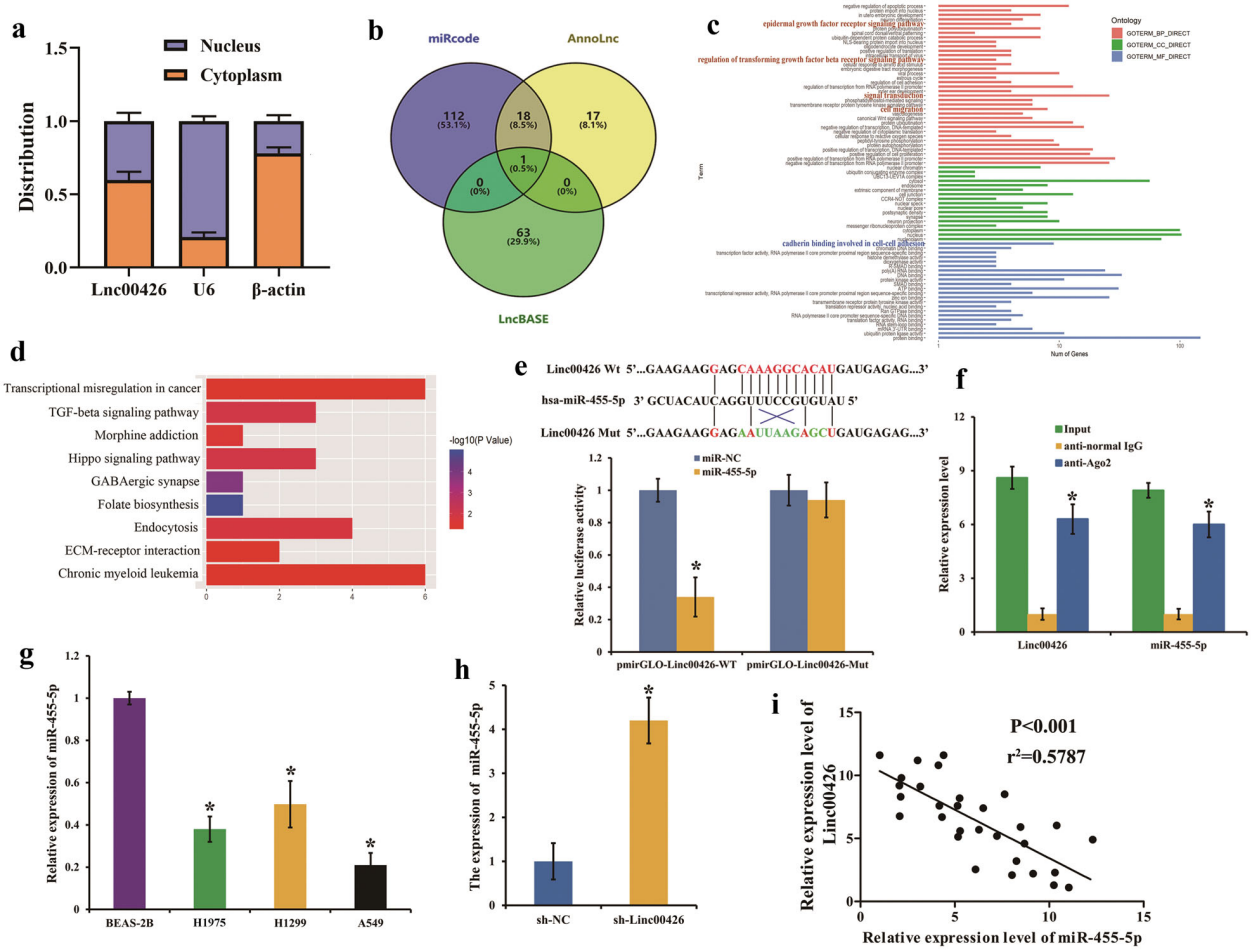
Linc00426 serves as a ceRNA by competitive binding to miR-455-5p. **a** Distribution of Linc00426 in A549 cells examined by RT-qPCR. U6 and GAPDH act as nuclear and cytoplasmic internal reference, respectively. **b** Overlap of miRcode, AnnoLnc, and LncBASE database. **c** GO analyses. **d** KEGG pathway analyses. **e** Predicted Linc00426 binding sites of the human miR-455-5p gene, with the corresponding sequence in the mutated version. Luciferase reporter gene assays are used to prove the interaction between miR-455-5p and Linc00426. Student’s *t* test. **f** RIP assay. Relative expression levels of Linc00426 and miR-455-5p examined by RT-qPCR. Student’s *t* test. **g** Expression of miR-455-5p tested in indicated cells by RT-qPCR. One-way ANOVA. **h** Expression of miR-455-4p in A549 cells with shNC and sh-Linc00426 is detected by RT-qPCR. Student’s *t* test. **i** Correlation between Linc00426 and miR-455-5p in 30 LUAD tissues (*r*^*2*^ = 0.5787, *P* < 0.001). Spearman correlation analysis. Experiments were performed three times and data were presented as mean ± SD. ^*^P < 0.05.

LncRNAs differ in subcellular distributions, with some predominantly in the cytoplasm while others in the nucleus. Accumulating evidence shows that LncRNAs can interact with miRNAs competitively and regulate mRNA expression predominantly in the cytoplasm, thereby affecting tumorigenesis and metastasis. We measured the location of Linc00426 through RT-qPCR based on the cytoplasm and nuclear RNA of A549 cells, respectively. The results showed that Linc00426 was a cytoplasm-enriched LncRNA in A549 cells (Fig. 4a). Therefore, it might function as an endogenous molecular sponge to modulate miRNAs. To identify the putative functional properties of Linc00426 in the tumorigenesis of LUAD and its connection with miRNAs expression, we analyzed interested miRNAs that putatively bind to Linc00426 using bioinformatics (AnnoLnc, miRcode, and LncBase predicted v.2) tools. MiR-455-5p is considered to a consensus target gene (Fig. 4b). The GO analysis indicated that miR-455-5p affected a list of genes associated with cell migration, cancer-related signal transduction (epidermal growth factor receptor and canonical Wnt signaling pathways), and cadherin binding was involved in cell-cell adhesion from three aspects of biological process, cellular component, and molecular function (Fig. 4c). KEGG pathway analysis was performed to reveal the possible biological functions of miR-455-5p integrated signature. The top KEGG pathways enriched for the miR-455-5p targets were mainly associated with cancer-relevant pathways and transcriptional misregulation in cancer (Fig. 4d). Such pathways included chronic myeloid leukemia, Hippo, and TGF-beta ones. Subsequent bioinformatics analyses revealed a complementary sequence for miR-455-5p in Linc00426. Luciferase reporter assays were conducted to illustrate the binding of miR-455-5p to Linc00426. The overexpression of miR-455-5p abolished the luciferase activity of the Wt reporter vector, exclusive of the mutated reporter vectors (Fig. 4e). Further research using miR-455-5p was conducted. To identify the relationship between Linc00426 and miR-455-5p, we performed RIP assay. Levels of Linc00426 and miR-455-5p were higher in the anti-Ago2 group than those in the anti-normal IgG group (Fig. 4f). Furthermore, we analyzed the levels of miR-455-5p in LUAD cells, the results showed that the expression of miR-455-5p was significantly down-regulated in all LUAD cells compared with that in BEAS-2B (Fig. 4g). The effects of Linc00426 knockdown on miR-455-5p expression were also examined in LUAD cells. MiR-455-5p was notably up-regulated after transfection of sh-Linc00426 into A549 cells (Fig. 4h). Our findings also suggested an inverse correlation between Linc00426 and miR-455-5p (Fig. 4i). Linc00426 functions as an endogenous molecular sponge to down-regulate miR-455-5p by competitive binding to miR-455-5p.

### Linc00426 acts as a ceRNA by sponging miR-455-5p and modulating UBE2V1

**Fig. 5:**
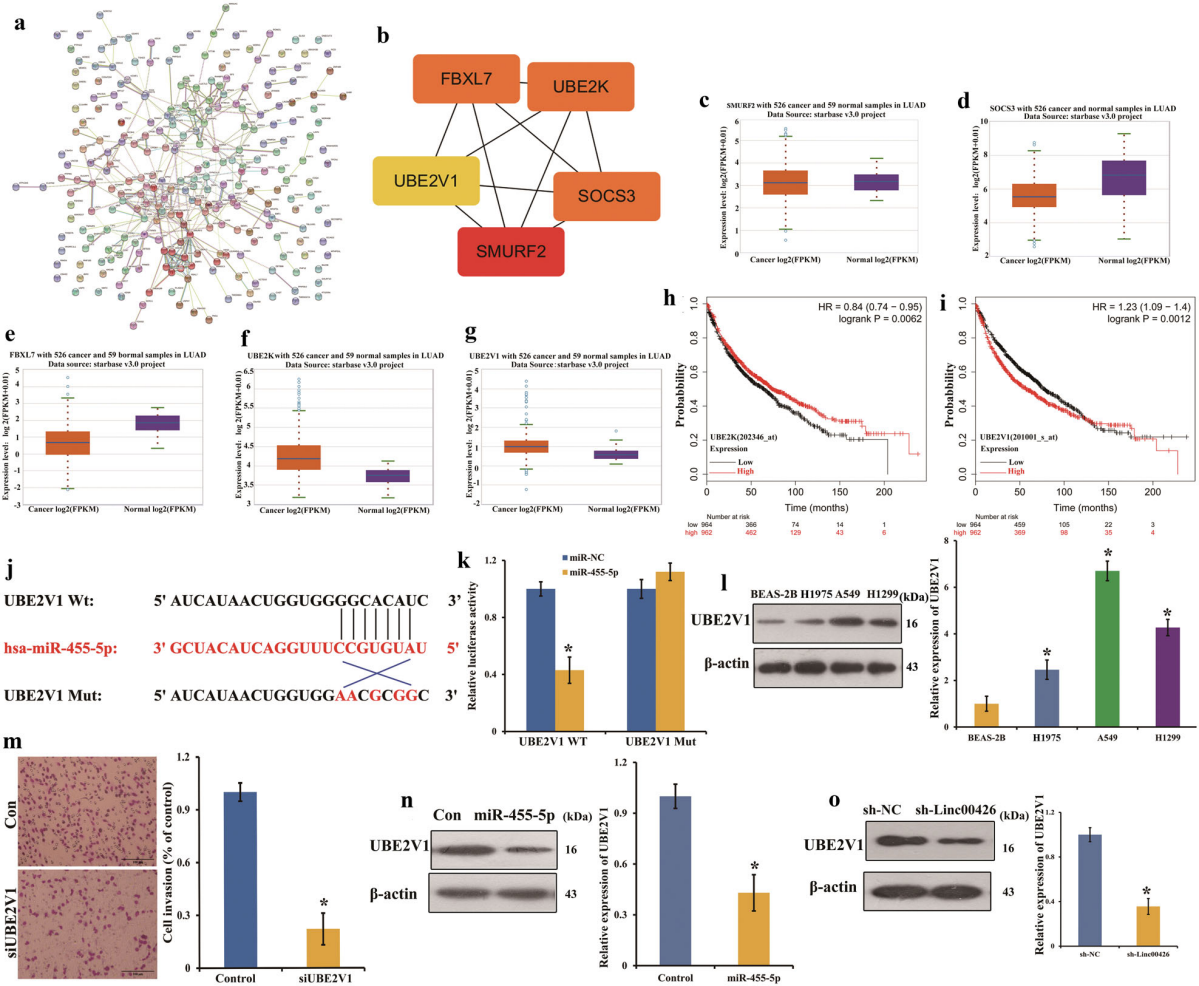
Linc00426 serves as a ceRNA by sponging miR-455-5p and modulating UBE2V1. **a** PPI network of targeted genes. **b** Hub genes of targeted genes. **c**–**g** Expression of hub genes in LUAD and normal lung tissues. **h**, **i** Kaplan–Meier plots of UBE2K and UBE2V1. **j** The UBE2V1 3′-UTR WT and UBE2V1 3′-UTR Mut. **k** The luciferase activity of transfected cells. Student’s *t* test. **i** The expression of UBE2V1 in LUAD cells and BEAS-2B cells is determined by Western blot. One-way ANOVA. **m** Effects of UBE2V1 on the invasive ability of A549 cells with control and siUBE2V1. Scale bar = 100 μm. Student’s *t* test. **n** Effects of miR-455-5p on protein levels of UBE2V1. Student’s *t* test. **o** Effects of sh-Linc00426 on protein levels of UBE2V1. Student’s *t* test. Experiments were performed three times and data were presented as mean ± SD. ^*^P < 0.05.

It has been found that one of the molecular mechanisms by which LncRNAs regulate gene expression is to interact with miRNA as ceRNAs that bind to miRNA response elements (MREs) and protect miRNAs from binding to and repressing target RNAs^20^. Using bioinformatics analysis (TargetScan) tools, we found that miR-455-5p was putatively bound to 258 targeted genes. Then, we used PPI analysis and cytoHubba algorithm of cytoscape to detect the potential targets for miR-455-5p. And the results revealed 5 genes of SMAD specific E3 ubiquitin protein ligase 2 (SMURF2), suppressor of cytokine signaling 3 (SOCS3), ubiquitin-conjugating enzyme E2 K (UBE2K), F-box, leucine rich repeat protein 7 (FBXL7) and UBE2V1 as the hub ones (Fig. 5a, b). Then, the results of starbase showed that UBE2K and UBE2V1 had higher expression in LUAD tissues (Fig. 5c–g). Kaplan–Meier plots showed that higher expression of UBE2V1 corresponded to a lower overall survival probability for LUAD patients. However, UBE2K had a higher overall survival probability (Fig. 5h, i). Therefore, we focused on UBE2V1. To verify the potential interaction between miR-455-5p and UBE2V1 in LUAD cells, we cloned the wild-type or mutant UBE2V1 3′-UTR downstream of the luciferase reporter gene (Fig. 5j). The plasmids were co-transfected with miR-455-5p into HEK-293T cells. The dual-luciferase reporter assay revealed that miR-455-5p was bound to UBE2V1 (Fig. 5k). We determined the expression of UBE2V1 in BEAS-2B and LUAD cells (H1975, A549, and H1299) by Western blot analyses. The results showed that the expression of UBE2V1 was significantly up-regulated in all LUAD cells compared with that in BEAS-2B cells (Fig. 5l). The invasion assay elucidated that UBE2V1 knockdown dramatically depressed cell invasion of A549 (Fig. 5m). These data suggest that UBE2V1 is an oncogene in LUAD. Meanwhile, the Western blot results clarified that overexpression of miR-455-5p markedly reduced the protein levels of UBE2V1 (Fig. 5n). Previous studies reported that LncRNAs can modulate many downstream genes to affect the biological functions of tumor cells^21,22^. We further investigated whether UBE2V1 is the downstream protein molecule for Linc00426. As predicted, the knockdown of Linc00426 reduced the expression of UAB2V1 in A549 cells compared with sh-NC (Fig. 5o).

### MiR-455-5p reverses the promoting effects of Linc00426 on the invasion and EMT of LUAD cells

**Fig. 6:**
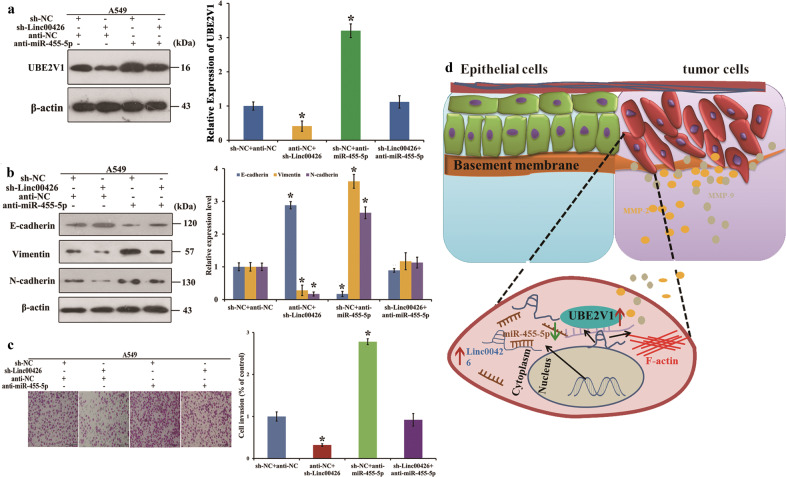
Linc00426 exerts invasion activities by inhibiting miR-455-5p and up-regulating UBE2V1 expression. **a** The expression of UBE2V1 in different cell groups. **b** The expression of EMT markers in different cell groups. **c** The invasion ability in different cell groups. Scale bar = 100 μm. Experiments were performed three times and data were presented as mean ± SD. ^*^P < 0.05. One-way ANOVA. **d** Schematic diagram for Linc00426-based regulatory mechanisms in LUAD cells.

We further detected relevant regulatory relationship among Linc00426, miR-455-5p, and UBE2V1. The group of sh-NC + anti-miR-455-5p enhanced the UBE2V1 level, while, sh-Linc00426+anti-NC significantly down-regulated the UBE2V1 level, which could be partially rescued by sh-Linc00426+anti-miR-455-5p (Fig. 6a). Figure 6b indicates that compared with sh-NC + anti-NC, sh-Linc00426+anti-NC suppresses the expression of Vimentin and N-cadherin but elevates that of E-cadherin. Moreover, sh-NC + anti-miR-455-5p elevate the expression of Vimentin and N-cadherin but suppress that of E-cadherin, which can be reversed by sh-Linc00426+anti-miR-455-5p. And down-regulated Linc00426 could suppress the EMT process stimulated by anti-miR-455-5p in A549 cells. In addition, we found that anti-miR-455-5p promoted the invasion ability of A549 cells, which could be reduced by down-regulated Linc00426. And the pro-invasive effect was contracted by co-transfected with anti-miR-455-5p (Fig. 6c). Collectively, these results suggest that Linc00426 accelerates LUAD progression by acting as a molecular sponge to regulate miR-455-5p (Fig. 6d).

## Discussion

LUAD is the most malignant subtype of NSCLC. Its incidence and mortality have being increased significantly^23^. In recent years, the ectopic expression of LncRNAs has drawn increased attention in cancer research since it plays an essential part in the proliferation, metastasis, and invasion of malignant tumors. Although numerous LncRNAs have been found in LUAD, few further researches have been conducted.

LncRNAs, transcripts with lengths ranging from 200 nt to 100 kb and widely distributed in the whole genome, do not encode proteins but modulate the expression of coding genes or non-coding RNAs^24,25^. LncRNAs were initially regarded as ineffective junk products. At present, they are considered to participate in vital regulatory functions, boosting yet another layer of complexity to our understanding of genomic modulation^26^. The diverse LncRNAs have been considered correlated with cell growth, cell apoptosis and epigenetic modulation, transcription, and translation in a particular spatiotemporal manner^27^. They are transcribed by RNA polymerase II, with some of them further regulated via splicing at the 5′ and 3′ ends and exported to the cytoplasm. Lnc-31 can control post-transcriptional gene expression which is required for myoblast proliferation and accelerate Rho-associated c, oiled-coil containing protein kinase 1 (ROCK1) protein synthesis by stabilizing its translational activator, Y-box binding protein 1 (YB-1)^28^. LncRNAs located in the cytoplasm could affect the mRNA level through ceRNAs. Diverse LncRNAs function as ceRNAs to adjust the EMT progression, such as LncRNA of HULC in hepatocellular carcinoma and LncRNA of H19 in colorectal cancer^29,30^. Linc00673 serves as a miR-150-5p sponge to adjust zinc finger E-box binding homeobox 1 (ZEB1) expression, thereby influencing the proliferation, migration, invasion, and EMT of NSCLC^31^. Our study reported the upregulation of Linc00426 (ENST00000417079), a 1045 nt intergenic non-coding RNA located at chromosome13:30, 340, 270-30, 373, and 899, in the majority of patients with LUAD. Its knockdown inhibits cell growth, invasion, metastasis, and F-actin expression, suggesting its vital role in the development and progression of LUAD.

With the development of bioinformatics technology, the genomics, transcriptome, and epigenomics of cancers were investigated^32^. It was reported that Linc00426 expression level is elevated in doxorubicin resistant osteosarcoma and significantly correlated with unfavorable prognosis^33^. In the present study, we identified Linc00426 highly expressed in LUAD by microarray, suggesting Linc00426 may function as an extensive tumor promoter. Therefore, elucidating its involvement in LUAD tumorigenesis needs further verification.

LncRNAs and miRNAs often regulate each other by a ceRNAs-related mechanism. In the present study, miR-455 could serve as an anti-oncogene in NSCLC through upregulation of ZEB1 and a potential therapeutic target for NSCLC^34^. Lan et al^35^. found that Serine/threonine kinase 17b (STK17B) promotes the EMT process via activating AKT/GSK-3β/Snail signal pathway, and miR-455-3p has been identified as an upstream regulator of STK17B. Circ0007142-miR-455-5p-SGK1 axis regulates cell proliferation, apoptosis, migration, and invasion of colorectal cancer cells^36^. It was reported that miR-455-5p suppresses prostate cancer cellular proliferation and induces cell apoptosis by downregulating C-C Motif Chemokine Receptor 5 (CCR5)^37^. LncRNA of Gm4419 accelerates hepatic I/R injury by targeting the miR-455 and SRY-Box transcription factor 6 (SOX6) axis^38^. Zhang et al.^39^ revealed a novel mechanism underlying MCM3AP-AS1 induced hepatocellular carcinoma metastasis by regulating miR-455. Consequently, in our study, Linc00426 and UBE2V1 are significantly up-regulated when miR-455-5p is knocked down. Knockdown of Linc00426 inhibits cell migration, invasion, and EMT. MiR-455-5p could reverse the biological function of Linc00426 in A549 cells.

UBE2V1 encodes Ubiquitin Conjugating Enzyme E2V1, a ubiquitin-conjugating E2 enzyme variant (UEV) protein. UEVs are similar in sequence to ubiquitin-conjugating E2 enzymes but lack their enzymatic activity^40^. UBE2V1 plays an important role in protein aggregate formation. Inhibition of UBE2V1 decreases aggregate formation through enhanced ubiquitin proteasome system performance rather than autophagy, and may provide a novel therapeutic target for treating cardiac proteinopathies^41^. UBE2V1 has been demonstrated as one of the key components of TRAF6 to control NF-κB activation^42^. Nevertheless, the role of UBE2V1 in cancers including LUAD and the mechanisms involved are still largely unknown. Our study proved that Linc00426 could sponge miR-455-5p and adjust the expression of UBE2V1 indirectly.

Indeed, there are some limitations in the present work. First, the microarray analyses were performed in only two groups of samples, which limits further bioinformatics analyses and the search for other mechanisms of Linc00426 underlying LUAD progression. Second, A549 cells are good representative LUAD cells. Nonetheless, more representative cell lines should be employed to validate our results. Finally, the animal experiment and the clinical study were performed to confirm the role of Linc00426 in LUAD. We did not study whether miR-455-5p could abolish the function of UBE2V1 in vivo. In future studies, we will examine the effects of miR-455-5p inhibition in Linc00426 knockdown cells in vivo and investigate other mechanisms by which Linc00426 plays the role as an oncogene in LUAD. Collectively, these results highlight the biomarker potential of Linc00426-miR-455-5p-UBE2V1 expression in LUAD, although it needs to be confirmed in a larger scale of independent samples.

To conclude, this research mainly focuses on the novel lncRNAs involving in proliferation, invasion, metastasis, and EMT of LUAD cells. We identified Linc00426 by screening the expression profile of lncRNAs in LUAD tissues with or without lymph node metastasis. Then we discovered significantly up-regulated Linc00426 in LUAD tissues and cell lines. Animal experiments revealed Linc00426 promotes tumorigenicity and metastasis in vivo. The cell biological experiments uncovered that Linc00426 serves as a ceRNA with miR-455-5p and regulates the expression of UBE2V1 in LUAD cells. Thus, our findings suggest the novel lncRNA of Linc00426 as a tumor promoter and potential therapeutic biomarker for LUAD.

### Supplementary information

Supplementary Materials

## Data Availability

The dataset supporting the conclusions of this article is included within the article.
