# Author Correction: Linc00426 accelerates lung adenocarcinoma progression by regulating miR-455-5p as a molecular sponge

**DOI:** 10.1038/s41419-024-06952-8

**Published:** 2024-08-05

**Authors:** Hongli Li, Qingjie Mu, Guoxin Zhang, Zhixin Shen, Yuanyuan Zhang, Jun Bai, Liping Zhang, Dandan Zhou, Quan Zheng, Lihong Shi, Wenxia Su, Chonggao Yin, Baogang Zhang

**Affiliations:** 1 Experimental Center for Medicine Research, Weifang Medical University, 261053 Weifang, China; 2 Department of Pathology, School of Clinical Medicine, Weifang Medical University, 261053 Weifang, China; 3 School of Clinical Medicine, Weifang Medical University, 261053 Weifang, China; 4 College of Biological Science and Technology, Weifang Medical University, 261053 Weifang, China; 5 Department of Clinical Surgery, Affiliated Hospital of Weifang Medical University, 261053 Weifang, China; 6 College of Nursing, Weifang Medical University, 261053 Weifang, China

Correction to: *Cell Death and Disease* 10.1038/s41419-020-03259-2, published online 11 December 2020

In this article in Figure 2F, we examined raw data and records from all experiments and found that the results for E-cadherin in Figure 2F were misused. Therefore, we want to replace these two misused images. The corrected versions of these images, ensure the accuracy of the data presented. This correction does not alter any results or conclusions of our paper. We apologize for any confusion and appreciate the community’s understanding.
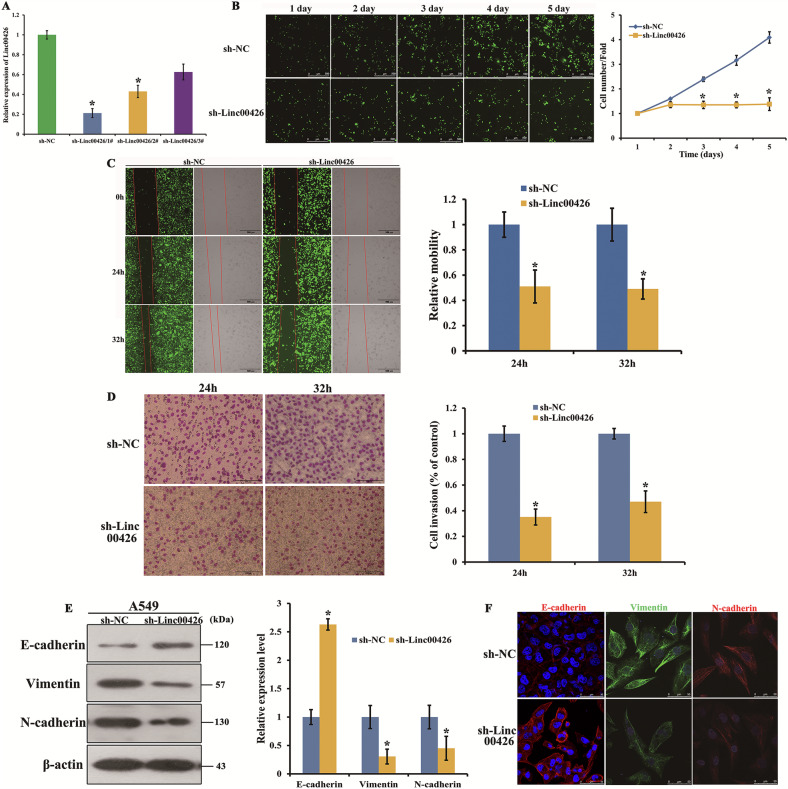

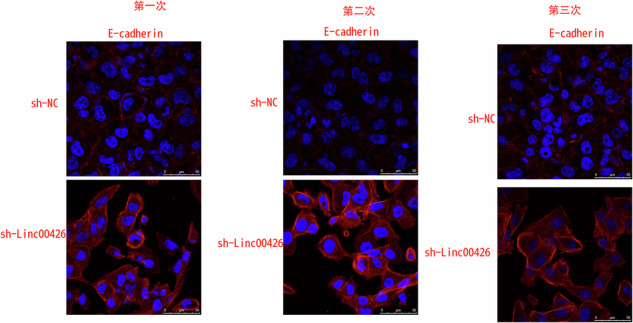


The original article has been corrected.

