# Supplementary material for: Linc00426 accelerates lung adenocarcinoma progression by regulating miR-455-5p as a molecular sponge
Source: Cell Death Dis. 2020 Dec 11;11(12):1051. doi: 10.1038/s41419-020-03259-2 (PMC7732829; doi:10.1038/s41419-020-03259-2)
Supplement: Supplementary file 1 — Supplementary Materials [file 41419_2020_3259_MOESM1_ESM.docx]

**Supplementary Table 1: Array datas of choose seven** **upregulated lncRNAs differentially expressed between LUAD tissues with lymph node metastasis and LUAD tissues without lymph node metastasis.**

| seqname | GeneSymbol | RNA length | Chromosome localization | Fold-change | lncRNA type |
| --- | --- | --- | --- | --- | --- |
| ENST00000417079 | LINC00426 | 1045 | chr13 | 3.9077832 | intergenic |
| ENST00000508260 | RP11-823P9.1 | 1012 | chr5 | 3.8873289 | intergenic |
| uc010fkx.1 | WASH2P | 1047 | chr2 | 3.8493264 | intergenic |
| ENST00000447250 | RP4-625H18.2 | 1016 | chr6 | 3.3828508 | intergenic |
| ENST00000421599 | SNHG11 | 1046 | chr20 | 2.671271 | intergenic |
| NR_034178 | SRGAP2B | 1033 | chr1 | 2.5732395 | intergenic |
| TCONS_00013943 | XLOC_006588 | 1031 | chr7 | 2.2628078 | intergenic |
